# Antibacterial Effect of Eugenol on *Shigella flexneri* and Its Mechanism

**DOI:** 10.3390/foods11172565

**Published:** 2022-08-25

**Authors:** Xiangyang Bai, Xuejiao Li, Xue Liu, Zeyu Xing, Ruiying Su, Yutang Wang, Xiaodong Xia, Chao Shi

**Affiliations:** 1 College of Food Science and Engineering, Northwest A&F University, Xianyang 712100, China; 2 School of Food Science and Technology, National Engineering Research Center of Seafood, Dalian Polytechnic University, Dalian 116304, China

**Keywords:** *Shigella flexneri*, eugenol, antibacterial activity, reactive oxygen species, contaminated food media

## Abstract

*Shigella flexneri* (*Sh. flexneri*), which can be found in food and the environment, is a widespread food-borne pathogen that causes human diarrhea termed “shigellosis”. In this study, eugenol, a natural active substance, was investigated for its antibacterial activity against *Sh. flexneri*. The minimum inhibitory concentration (MIC) and minimum bactericidal concentration (MBC) of eugenol against *Sh. flexneri* ATCC 12022 was 0.5 and 0.8 mg/mL. The growth curves and inhibitory effect in LB broth, PBS, vegetable juice, and minced pork showed that eugenol had a good activity against *Sh. flexneri*. Research findings indicated the superoxide dismutase activity of *Sh. flexneri* was inhibited after eugenol treatment, resulting in concentrations of intracellular reactive oxygen species and an increase in malondialdehyde. The flow cytometry analysis and field emission scanning electron microscopy results revealed obvious damage to cell membrane integrity and changes in the morphology of *Sh. flexneri*. In addition, the intracellular ATP concentration leaked from 0.5 μM to below 0.05 μM and the membrane potential showed a concentration-dependent depolarization after eugenol treatment. In summary, eugenol exerted strong antibacterial activity and has the potential to control *Sh. flexneri* in the food industry.

## 1. Introduction

*Shigella flexneri* (*Sh. flexneri*), which is a rod-shaped, non-spore forming, non-flagella, facultative anaerobic Gram-negative bacterium [1], has been confirmed to be widespread in vegetable salads, meat, raw milk, sea food and other products [2]. In addition, as it tends to spread rapidly in closed and concentrated groups, *Sh. flexneri* has been detected in schools, restaurants, and other environments [3]. *Sh. flexneri* can cause bacillary dysentery with clinical symptoms as painful abdominal cramps, nausea, fever, and even colitis [4]. Dysentery caused by *Sh. flexneri* is considered to be the most common explosive disease in developing countries [5], which has led to 1.7 million episodes of bacillary dysentery in China per year. Additionally, it has become one of the major causes of morbidity and mortality in children with diarrhea in developing countries [6]. Furthermore, the high survival rate and strong tolerance of *Sh. flexneri* makes it able to survive for extended periods under adverse conditions, such as high acidity or high temperatures. What is more, it can even remain viable following several hours of exposure at pH 2–3 [7]. Thus, the tolerance of *Sh. flexneri* to low-pH conditions may enhance survival in acidic foods and also in the acidic environment of the human stomach, thereby increasing the potential for causing infection [8].

The control of *Sh. flexneri* in food has usually adopted sterilization methods, such as heat sterilization, ultra-high pressure, UV, or adding antibacterial substances to achieve the aim of inhibiting pathogens [9,10]. For the organic acid sterilization, studies have shown that in response to environmental encounters with acid, *Sh. flexneri* has evolved complex, inducible acid survival strategies [11]. In the process of food storage and sales, there are also ways to use food preservatives for retarding the bacterial spoilage of fish to keep products fresh [12]. However, use of food preservatives or antibiotics is limited, which, to a large extent, depends on their potential toxicity problems and their impact on food quality [13]. In general, for traditional sterilization methods such as heat sterilization, ultra-high pressure, and radiation treatment, they will be restricted by cost and equipment factors and may cause adverse effects on food quality and sensory [14,15]. Therefore, looking for efficient, safe, low-cost sterilization methods that do not affect food quality and sensory characteristics is of great significance for the control of *Sh. flexneri*.

In recent years, researchers have focused on the antibacterial activity of widely-sourced natural, plant-derived active substances. These natural substances are safe and nutritious [16], which has led to their widespread attraction for antibacterial applications in food [17]. Eugenol (C_10_H_12_O_2_; 4-allyl-2-methoxyphenol), the major ingredient in clove oil, is an aromatic compound belonging to the group of phenols, which is a pale-yellow liquid with a strong clove aroma and a mild spicy aroma [18]. It naturally exists in the leaves of *Laurel*, *Syringa* plants, clove oil, bay leaf oil, and other plants and essential oils [18], which can be used as a food additive or a dietary supplement, and its safety has been approved by the Food and Drug Administration (FDA) [17]. Also, studies have shown that eugenol is an effective antioxidant with high active oxygen scavenging ability that can enhance the antioxidant status of cells and prevent cell oxidative damage [19]. The antibacterial activity of eugenol has also attracted more and more attention. Research by Devi et al. [20] showed that eugenol has a strong inhibitory effect on the growth of S. Typhimurium. What’s more, research by Matan et al. [21] showed that eugenol has an inhibitory effect on *Aspergillus niger* in food. However, the antibacterial effect and mechanism of eugenol against *Sh. flexneri* have not been investigated.

Therefore, the objective of the present study was to evaluate the antibacterial activity that eugenol displays against *Sh. flexneri* by measuring the minimum inhibitory concentration (MIC), minimum bactericidal concentration (MBC), growth curves, and the inhibitory effect of eugenol on *Sh. flexneri* in nutrient and non-nutrient media. Subsequently, the superoxide dismutase (SOD) activity, intracellular reactive oxygen species (ROS) level, malondialdehyde (MDA) concentration, ATP concentration, membrane potential, membrane permeability, and cell morphology were assessed to explore the underlying mechanisms. In addition, the inactivation activity of eugenol on *Sh. flexneri* in minced pork and vegetable juice was investigated, which may provide a theoretical basis for eugenol as an antibacterial agent in controlling *Sh. flexneri* contamination in the food processing chain.

## 2. Materials and Methods

### 2.1. Reagents

Eugenol (HPLC ≥ 99%, CAS number 97-53-0) was acquired from Mansite Biological Technology Co. (Chengdu, China) and was dissolved in dimethyl sulfoxide (DMSO). The final concentration of DMSO in all sample solutions was 0.5% (*v*/*v*). Luria–Bertani (LB) agar and LB broth were purchased from Land Bridge Technology Co., LTD. (Beijing, China). All other chemicals and reagents were of analytical grade.

### 2.2. Bacterial Strains and Culture Conditions

*Sh. flexneri* (strain no. 12022) was obtained from the American Type Culture Collection (ATCC, Manassas, VA, USA). The strain was activated by incubation on LB agar at 37 °C for 18 h. Subsequently, a single colony on LB agar was inoculated in LB broth and cultured at 37 °C for 18 h. *Sh. flexneri* cells were harvested by centrifugation at 8000× *g* and 4 °C for 5 min and washed twice with sterile phosphate-buffered solution (PBS, pH 7.2). The cell density of strain suspension was adjusted to a wavelength of 600 nm (OD_600_) of 0.5 (approximately 3 × 10^8^ CFU/mL). All bacterial media were purchased from Beijing Land Bridge Technology Co., LTD. (Beijing, China).

### 2.3. Determinations of MIC and MBC

These assays were determined using the broth microdilution method as recommended by the Clinical and Laboratory Standards Institute [22], with minor modifications. The bacterial suspension, which was prepared as described in Section 2.2, was diluted to approximately 5 × 10^5^ CFU/mL in LB broth. Eugenol was diluted in LB broth and was then mixed with an equal volume of bacterial cultures to obtain the final eugenol concentrations of 0, 0.03125, 0.0625, 0.125, 0.25, 0.5, 1, and 2 mg/mL. Two-hundred microliters of the mixed liquor were added into each well of the 96-well microtiter plate, after which samples were incubated at 37 °C for 24 h. The MIC was defined as the lowest eugenol concentration that bacteria after incubation were tested without producing any visible growth. To determine the MBC, 100 µL of bacterial solution from each well was plated on an LB agar plate and cultured for 48 h. MBC was defined as the lowest eugenol concentration that completely inhibited all bacteria.

### 2.4. Growth Curve Analysis

The determination of the effect of eugenol on the growth curve of *Sh. flexneri* was done according to the method of Bsbii et al. [23], with minor modifications. The activated *Sh. flexneri* was cultured as described in Section 2.2 above, and the bacterial suspension was diluted to 2 × 10^6^ CFU/mL by LB broth. LB broth dissolved in 0.5% DMSO and different concentrations of eugenol were mixed with equal volumes of bacterial suspension and vortexed, which made the final concentration of eugenol 0 (Control), 1/16MIC, 1/8MIC, 1/4MIC, 1/2MIC, MIC, or 2MIC, respectively. At the same time, the LB broth containing 0.5% DMSO was used as the blank background control. After 200 μL of the mixed solution was added to each well of the honeycomb plate, the growth rate was measured by an automated microbiology growth curve analyzer (Bioscreen C; Labsystems, Helsinki, Finland) at 600 nm at 2 h intervals, which was cultured at 37 °C for 24 h.

### 2.5. Inactivation Effect of Eugenol on Sh. flexneri in LB and PBS

The inactivation effect of eugenol on *Sh. flexneri* was performed according to the procedure describe by Gao et al. [24], with slight modifications. The bacterial suspension that was prepared according to the method from Section 2.2 was diluted to approximately 1 × 10^6^ CFU/mL in LB broth or PBS. Then, equal volumes of LB broth with eugenol dissolved in it were mixed with the individual samples to obtain final eugenol concentrations of 0 (Control), MIC, 3/2MIC, and 2MIC. Similarly, bacteria were treated with 0 (Control), 1/2MIC, MIC, 3/2MIC, and 2MIC eugenol in PBS. After being treated for 0, 0.5, 1, 2, 4, 6, and 8 h at 37 °C, the mixed bacterial suspension was spread on LB agar plates and incubated at 37 °C for 12 h. The total number of colonies of *Sh. flexneri* were expressed as log CFU/mL.

### 2.6. The Bactericidal Effect of Eugenol on Food Easily Contaminated by Sh. flexneri

#### 2.6.1. Inactivation Effect of Eugenol on *Sh. flexneri* in Vegetable Juice

The method for the inactivation effect of eugenol against *Sh. flexneri* in vegetable juice was carried out according to the method described by Yuan et al. [25], with modifications. The lettuce juice, which was sterilized by irradiation in advance, was incubated with *Sh. flexneri* to give a final concentration of 1 × 10^6^ CFU/mL. Then, eugenol solution was added to the individual samples to achieve the final eugenol concentrations of 0 (Control), MIC, 3/2MIC, and 2MIC, and samples were further placed at 25 °C. The number of *Sh. flexneri* ATCC 12022 treated by eugenol for 0, 0.5, 1, 2, 4, 6, and 8 h was enumerated by spreading the bacterial suspension on LB agar plates after a 12-h incubation at 37 °C.

#### 2.6.2. Inactivation Effect of Eugenol on *Sh. flexneri* in Minced Pork

In view of the method used by Sun et al. [26], the inhibitory effect of eugenol on *Sh. flexneri* in minced pork was explored. The pure lean pork was irradiated and sterilized, and then the cleaned pork was broken into minced meat and packed in homogeneous bags. The *Sh. flexneri* suspensions prepared in Section 2.2 were grown to an OD_600_ value of 0.5 in 0.1% (*v*/*v*) BPW. Next, the bacterial suspension (1 × 10^6^ CFU/mL) was added to the above homogeneous bags and the attachment of bacteria was completed. Then, the eugenol was added to the individual bacterial suspension bags so that the final concentration was 0 (Control), 5MIC, 10MIC, and 15MIC, respectively. After being mixed uniformly, homogeneous bags with mixed pork were placed in a 10 °C constant temperature incubator to simulate processing and storage. A small portion of the sample was removed from each mixture at 0, 1, 2, 4, 6, and 8 h, was weighed, and then was eluted with 1 mL of BPW to spread on LB agar plates, and finally, was incubated at 37 °C for 12 h. The viable bacterial cells of *Sh. flexneri* were counted after incubation, and colonies were enumerated and calculated as log CFU/g.

### 2.7. Mechanism of Antibacterial Effects

#### 2.7.1. Assays of ATP Concentrations

The method for determining the intracellular ATP concentration change described by Guo et al. [27] was followed. The bacterial suspension prepared according to the method in Section 2.2 was mixed with eugenol solution with different concentrations to make the final concentration of eugenol in the sample 0 (Control), MIC, 2MIC, and 4MIC, respectively. The negative control group was determined by adding different concentrations of eugenol to PBS. Both sets of samples were incubated at 37 °C for 30 min, then transferred to an ice bath and subjected to ultrasonic cell lysis one by one. After lysis, samples were placed at 100 °C for 3 min immediately, which deactivated the ATPase in the sample. The bacterial solution was centrifuged (10,000× *g*, 4 °C, 5 min) and the supernatant was stored in an ice bath. The supernatant (100 µL) was inoculated into a white 96-well microplate before prepared ATP detection reagent (100 µL) was added to each well and then the luminescence intensity of the mixture was detected by a multimode microplate reader platform (Spark^®^; Tecan, Männedorf, Switzerland). The ATP working curve was drawn by measuring the luminescence intensities of the ATP standard solutions with final concentrations of 0.01, 0.1, 1, and 10 µmol/L, so that the intracellular ATP concentration can be calculated.

#### 2.7.2. Measurement of Membrane Potential

With reference to the method of Guffey et al. [28], the effect of eugenol on the membrane potential of *Sh. flexneri* was determined with the help of DiBAC_4_(3) anionic fluorescent dye. The bacterial suspension (125 µL) was cultured according to method in Section 2.2 and was added into the black 96-well microplate and incubated at 37 °C for 30 min, after which the anionic fluorescent dye DiBAC_4_(3) was diluted to a final concentration of 1 µmol/L in each well. The different concentrations of eugenol diluent (125 µL) were added to the black microplate to make the final concentration of original bacterial suspension 0, MIC, 2MIC, and 4MIC, respectively. The fluorescence intensity was measured every 5 min at the excitation and emission wavelengths of 492 and 515 nm for 30 min. The background fluorescence resulting from the eugenol added to the PBS was determined and the results were corrected. The membrane potential was expressed as the relative fluorescence intensity of eugenol-treated cells compared to that of the control cells.

#### 2.7.3. Flow Cytometry Investigation

The bacterial cell membrane permeability was detected by staining with propidium iodide (PI) and SYTO 9, followed by analysis using a flow cytometer (CytoFLEX; Beckman, Brea, CA, USA) as described by Wang et al. [29], with some modifications. Briefly, the cell suspension (approximately 1 × 10^6^ CFU/mL) was cultured with eugenol (0, MIC, 2MIC, 4MIC) at 37 °C for 1 h. After treatment, the bacterial cells were collected by centrifugation at 10,000× *g* for 2 min at 4 °C and then resuspended in 200 μL of 0.85% (*m*/*v*) NaCl solution. Subsequently, the cells were incubated with 1 μL of an equal volume of SYTO 9 and PI mixed dye for 15 min in the dark. Then, the extent of cell membrane permeability was detected using a flow cytometer.

#### 2.7.4. Effect of Eugenol on Intracellular ROS Production

The level of intracellular ROS generation was estimated using 2′,7′-dichlorodihydrofluorescein diacetate (DCFH-DA; Beyotime Institute of Biotechnology, Shanghai, China) by the method of Li et al. [30]. Bacteria cultured in Section 2.2 were collected by centrifugation at 8000× *g* for 5 min at 4 °C and washed twice with PBS. After eugenol treatment (0, 1/2MIC, MIC, 2MIC) for 20 min, samples were incubated with DCFH-DA at a final concentration of 5 μmol/L for 15 min at 37 °C in the dark. The blank background control was established by a combination of PBS and DCFH-DA without any pathogens. The strained cells were collected by centrifugation at 12,000× *g* for 10 min, then washed with PBS twice. The fluorescence was measured with a multimode microplate reader platform (Spark^®^; Tecan, Männedorf, Switzerland) at excitation and emission wavelengths of 488 and 525 nm, respectively. Aliquots taken from each mixture were placed onto LB agar plates to determine the viable bacterial number. The fluorescence intensity of the blank background control was subtracted from that of the treated cells and the data were normalized against the viable bacterial number.

#### 2.7.5. SOD Activity

The effects of eugenol on SOD in *Sh. flexneri* were studied according to Shi et al. [31], with minor modifications. The cells were harvested after being cultured according to Section 2.2 and were treated with PBS (Control) or eugenol (1/2MIC, MIC, 2MIC) at 37 °C for 20 min. Before the lysozyme (Solarbio, Beijing, China) was lysed at 37 °C for 6 min, bacteria were harvested. Then, the supernatant was collected by centrifugation (10,000× *g*, 3 min, 4 °C). The NBT/enzyme and reaction start working solution were configured under the guidance of the manual. The NBT/enzyme working solution, the extracted supernatant, and the reaction initiation working solution were sequentially added to the wells, and the 96-well plate was incubated for 30 min at 37 °C, and then the absorbance was read at 560 nm using a microplate reader (Spark^®^; Tecan, Männedorf, Switzerland).

#### 2.7.6. Determination of Lipid Peroxidation

The micro-MDA assay kit (Beijing Solarbio Science & Technology, Beijing, China) was used for measuring the formation of MDA as previously described, with some modifications [32]. In brief, the bacteria were exposed to eugenol (0 (Control), 1/2MIC, MIC, 2MIC) for 20 min at 37 °C, were then harvested by centrifugation at 8000× *g* for 10 min, and then the supernatant was discarded. After the extracting solution (1 mL) was added to the individual samples, which were placed in an ice bath, the samples were subjected to ultrasound for 6 min under 3/6 s pulse on/off conditions one by one. After lysis, the supernatant and MDA detection working solution were mixed and heated at 100 °C for 10 min and then cooled, followed by centrifugation at 4 °C and 10,000× *g* for 10 min. The blank control group was established by a combination of the MDA detection working solution and ultra-pure water. The absorbance of the supernatant at 450 nm, 532 nm, and 600 nm was measured by a multimode microplate reader platform (Spark^®^; Tecan, Männedorf, Switzerland), and the result was calculated according to the formula below: where ∆OD_532_, ∆OD_600_, and ∆OD_450_ refer to the difference between the absorbance of the positive control group and blank control ground at 532, 600, and 450 nm, respectively.

(1) $$\mathrm{MDA}\;\mathrm{concentration}\;\left(\mathrm{nmol}\right)=5\times \left(12.9\times \left(\Delta {\mathrm{OD}}_{532}-\Delta {\mathrm{OD}}_{600}\right)-2.58\times \Delta {\mathrm{OD}}_{450}\right)$$

#### 2.7.7. Field Emission Scanning Electron Microscope (FESEM) Observations

FESEM was performed as described by Sun et al. [26], with some modifications. The bacterial suspension was treated with eugenol with concentrations of 0 (Control), MIC, 2MIC, and 4MIC, and then incubated at 37 °C for 2 h and 4 h, respectively. The cells were collected, centrifuged (5000× *g*, 5 min, 4 °C), and washed twice with PBS. The cell pellet was resuspended in sterile water containing 2.5% (*v*/*v*) glutaraldehyde and incubated overnight at 4 °C to fix the cells. After centrifugation, cells were eluted with a water–ethanol gradient (30%, 50%, 70%, 80%, 90%, 100%, *v*/*v*) for 10 min before being fixed on glass slides. Following gold spray treatment under vacuum, the cells were observed using a field-emission scanning electron microscope (S-4800; Hitachi, Tokyo, Japan).

### 2.8. Statistical Analysis

All assays were performed in triplicate and all experimental data were analyzed using the SPSS version 20.0 (SPSS Inc., Chicago, IL, USA). A difference analysis was performed using a least significant difference test and the results were expressed as the mean ± standard deviation. Significance was expressed as: *, *p* < 0.05; **, *p* < 0.01.

## 3. Results

### 3.1. MIC and MBC of Eugenol

**Figure 1:**
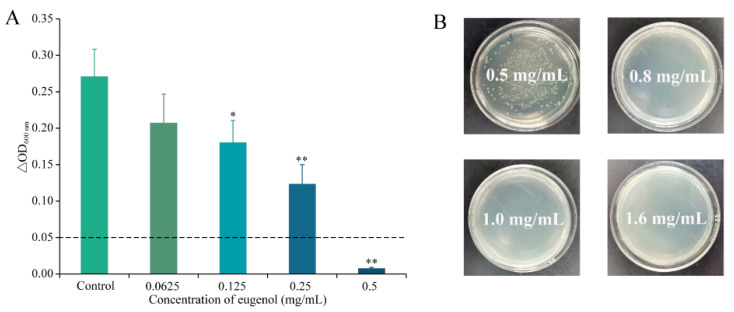
The MIC (**A**) and MBC (**B**) of eugenol against *Sh. flexneri* ATCC 12022. * indicates statistical significance of *p* < 0.05 compared with the control, ** indicates statistical significance of *p* < 0.01 compared with the control.

As shown in Figure 1A, when *Sh. flexneri* was treated with eugenol at 0.5 mg/mL, the ∆OD_600nm_ was less than 0.05. Therefore, the MIC value of eugenol was 0.5 mg/mL for *Sh. flexneri* ATCC 12022. As shown in Figure 1B, the samples at 0.5 mg/mL had bacterial growth on LB agar, while the samples at 0.8, 1.0, and 1.6 mg/mL had no colonies growing on LB agar, indicating that the bacteria were inactivated by eugenol. Therefore, the MBC was 0.8 mg/mL.

### 3.2. Effects of Eugenol on Sh. flexneri Growth Curve

**Figure 2:**
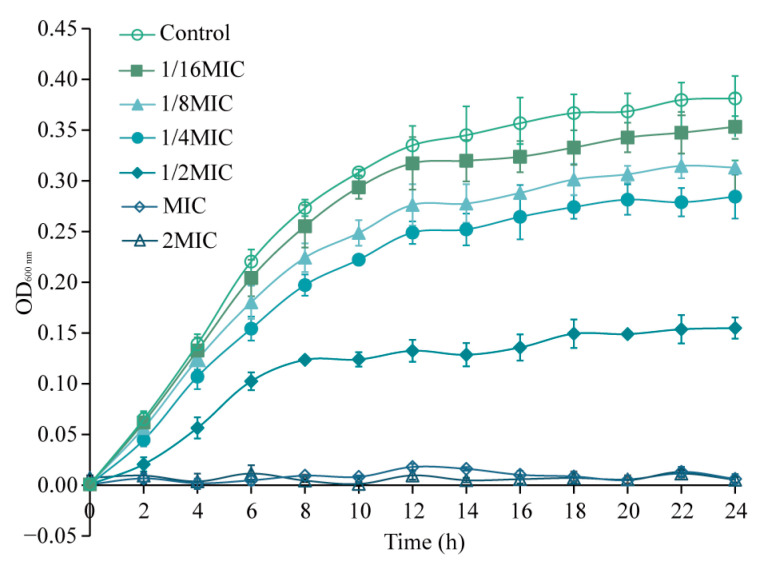
Growth curve analysis of *Sh. flexneri* with various concentrations of eugenol.

The results showed that the growth of *Sh. flexneri* was completely inhibited by eugenol at MIC and 2MIC (Figure 2). *Sh. flexneri* treated with eugenol at 1/2MIC showed an obvious reduction in growth when compared with the control, and the populations of *Sh. flexneri* treated with eugenol at 1/4MIC, 1/8MIC, and 1/16MIC were reduced compared with the control group.

### 3.3. Inactivation Effect of Eugenol on Sh. flexneri in LB and PBS

**Figure 3:**
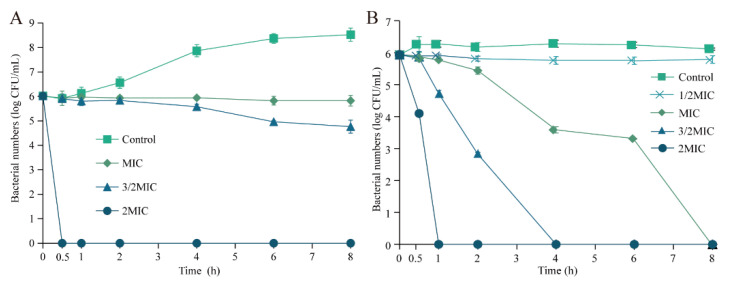
Inactivation effect of eugenol on *Sh. flexneri* in the LB broth (**A**) and PBS (**B**).

The inhibitory effects of eugenol on *Sh. flexneri* in LB and PBS were shown in Figure 3. The initial concentration of *Sh. flexneri* in the bacterial solution was about 6.0 log CFU/mL. The population of *Sh. flexneri* without eugenol treatment increased to 8.3 ± 0.1 log CFU/mL after 8 h. In the nutrient medium LB broth (Figure 3A), after 0.5 h, the population of *Sh. flexneri* decreased to below the detection limit (1 CFU/mL) after treatment with eugenol at 2MIC. In the non-nutrient medium PBS, the population of *Sh. flexneri* treated with eugenol at 2MIC, 3/2MIC, and MIC fell below the detection limit at 1 h, 4 h, and 8 h, respectively (Figure 3B).

### 3.4. The Bactericidal Effect of Eugenol on Food Easily Contaminated by Sh. flexneri

#### 3.4.1. Inactivation Effect of Eugenol on *Sh. flexneri* in Vegetable Juice

**Figure 4:**
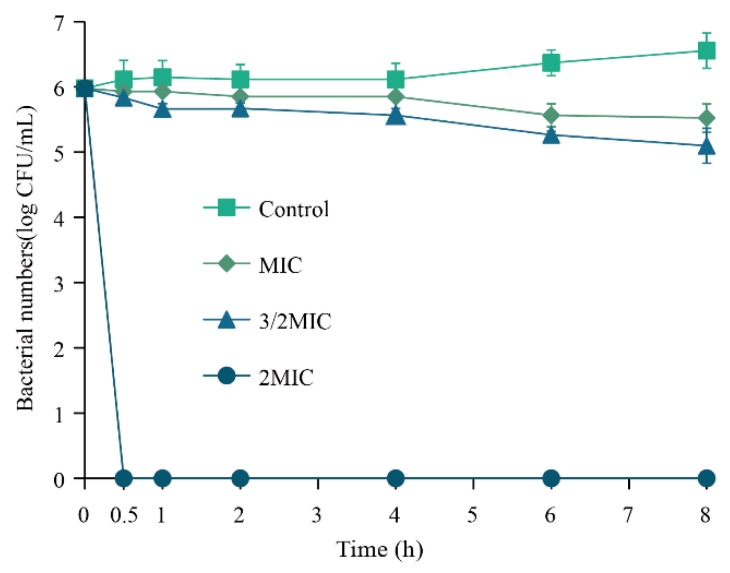
Inactivation effect of eugenol on *Sh. flexneri* in vegetable juice.

The survival of *Sh. flexneri* treated with eugenol in lettuce juice (Figure 4) was examined. The initial concentration of the bacterial solution was about 6.0 log CFU/mL. The population of *Sh. flexneri* without eugenol treatment increased to 6.6 ± 0.1 log CFU/mL after 8 h. After 8 h, the population of *Sh. flexneri* treated with eugenol at MIC and 3/2MIC decreased by 5.52 ± 0.21 and 5.10 ± 0.26 log CFU/mL, which was close to the untreated control. However, the number of *Sh. flexneri* treated with eugenol at 2MIC dropped to below the limit of detection (1 CFU/mL) after 0.5 h.

#### 3.4.2. Inactivation Effect of Eugenol on *Sh. flexneri* in Minced Pork

**Figure 5:**
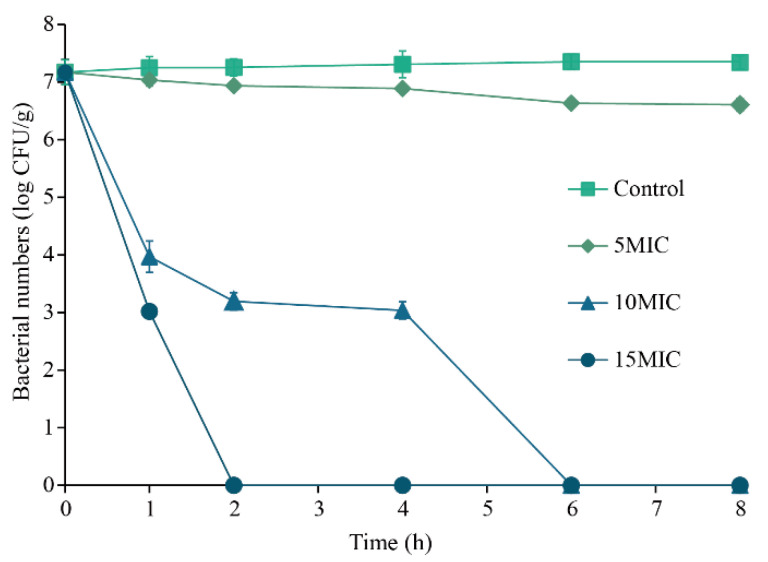
Inactivation effect of eugenol on *Sh. flexneri* in minced pork.

The antibacterial effects of eugenol on *Sh. flexneri* in ground pork were shown in Figure 5. The initial concentration of the bacterial solution was about 7.2 ± 0.2 log CFU/g. The population of *Sh. flexneri* without eugenol treatment increased to 7.3 ± 0.1 log CFU/g after 8 h. The samples at 5MIC decreased the concentrations of bacteria to about 6.6 ± 0.1 log CFU/g after being treated for 6 h. However, the population of *Sh. flexneri* treated with eugenol at 10MIC and 15MIC fell below the detection limit at 2 h and 6 h, respectively.

### 3.5. Mechanism of Antibacterial Effects

#### 3.5.1. Effects of Eugenol on Intracellular ATP Concentration of *Sh. flexneri*

**Figure 6:**
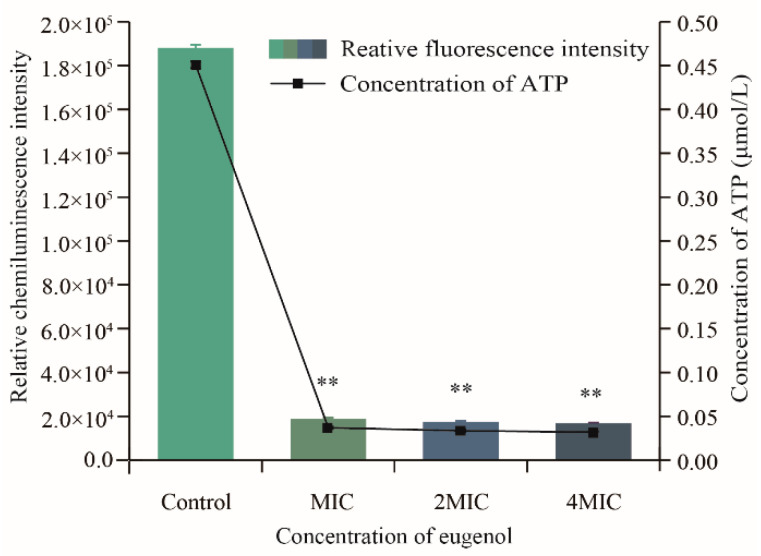
The effects of eugenol on intracellular ATP concentration of *Sh. flexneri*. ** indicates statistical significance of *p* < 0.01 compared with the control.

The effects of eugenol on intracellular ATP concentrations of *Sh. flexneri* ATCC 12022 were shown in Figure 6. There was a good linearity between the relative luminescence units and ATP concentration (y = 408,791x + 3732.4, R^2^ = 0.9999). Compared with the control, the intracellular ATP concentrations of the bacteria were significantly reduced after being treated with eugenol. The intracellular ATP concentration of bacteria in the control was 0.451 ± 0.004 μM, while the intracellular ATP concentrations of the eugenol-treated bacteria (MIC, 2MIC and 4MIC) were significantly reduced (*p* < 0.01) to below 0.05 μM.

#### 3.5.2. Effects of Eugenol on Cell Membrane Potential of *Sh. flexneri*

**Figure 7:**
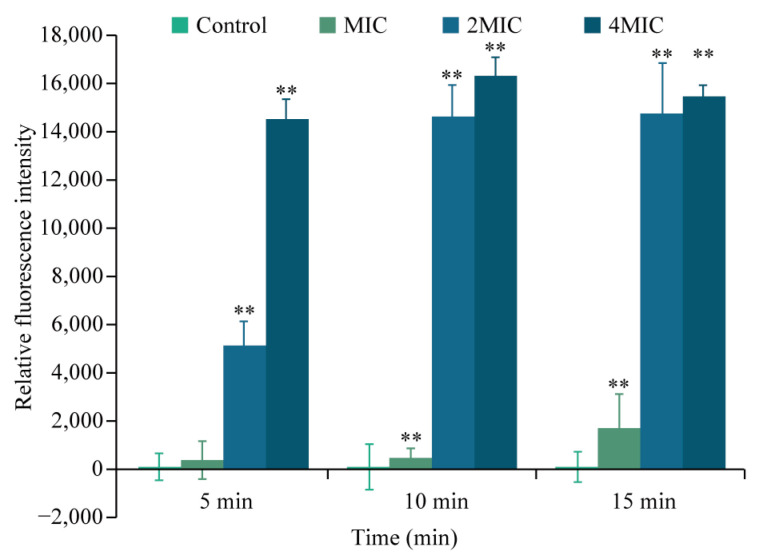
Changes in cell membrane potential of *Sh. flexneri* treated with eugenol. ** indicates statistical significance of *p* < 0.01 compared with the control.

As shown in Figure 7, the membrane potential of *Sh. flexneri* cells treated with eugenol indicated membrane depolarization as a result of an increase in membrane potential compared with the control, and the degree of depolarization increased with the increase in eugenol concentrations and the incubation time during those 5–10 min.

#### 3.5.3. Effects of Eugenol on Cell Membrane Integrity of *Sh. flexneri*

**Figure 8:**
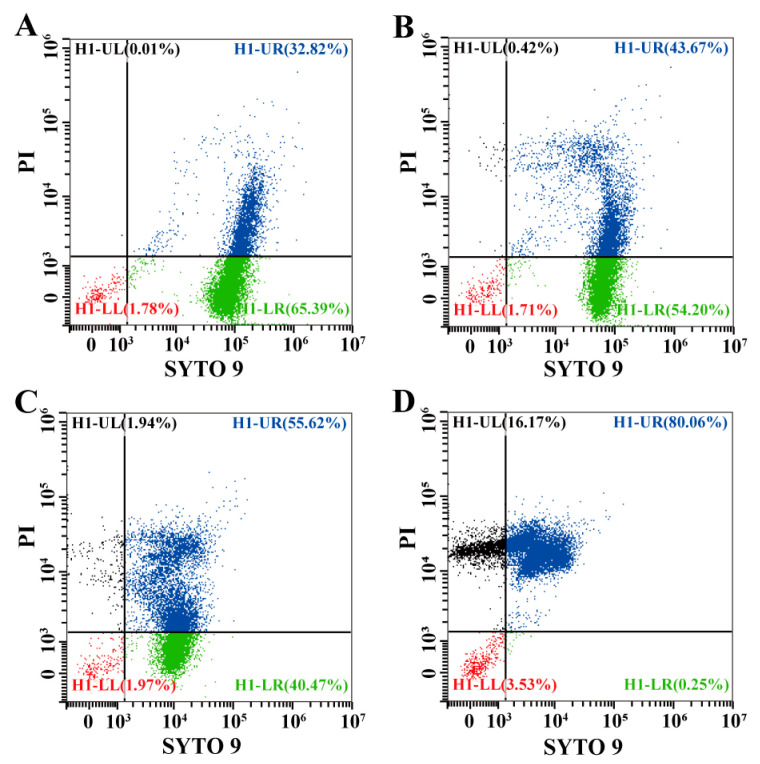
The effects of eugenol on the membrane integrity of *Sh. flexneri* by flow cytometry. (**A**) Untreated *Sh. flexneri* cells. (**B**) *Sh. flexneri* treated with eugenol at MIC. (**C**) *Sh. flexneri* cells treated with eugenol at 2MIC. (**D**) *Sh. flexneri* cells treated with eugenol at 4MIC.

The changes in the cell membrane integrity of *Sh. flexneri* were detected by flow cytometry, which is often used for the qualitive and quantitative detection of cells. Four quadrants were used to represent different cell states, with H1-LL, H1-LR, H1-UR, and H1-UL representing unstained bacteria, stained by SYTO 9 only, stained by SYTO 9 and PI, and stained by PI only, respectively. As shown in Figure 8, compared with the control, eugenol could decrease the SYTO 9-stained (H1-LR) rate of cells while increasing the rates of cells strained by SYTO 9 and PI simultaneously (H1-UR). The PI-labeled populations (H1-UL) in the 0 (Control), MIC, 2MIC, and 4MIC eugenol-treated groups were 0.01%, 0.42%, 1.94%, and 16.17%, respectively, which were recognized to increase with an increase in eugenol concentration.

#### 3.5.4. Effects of Eugenol on ROS Level in *Sh. flexneri*

**Figure 9:**
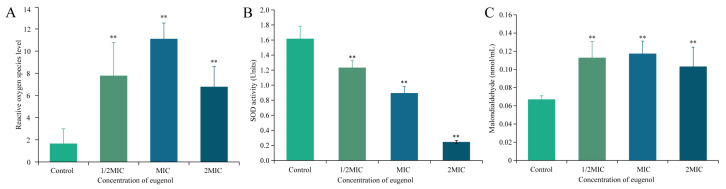
The effects of eugenol on intracellular ROS (**A**), SOD (**B**), and MDA (**C**) levels in *Sh. flexneri*. ** indicates statistical significance of *p* < 0.01 compared with the control.

The effects of eugenol on ROS levels of *Sh. flexneri* ATCC 12022 were shown in Figure 9A. The intracellular ROS levels of eugenol-treated samples were higher than the control, while the ROS level of the samples at 2MIC, which was also significantly higher than the control, was decreased compared with MIC. After treatment of *Sh. flexneri* with eugenol at 1/2MIC, MIC, and 2MIC, the intracellular ROS levels increased significantly (*p* < 0.01) to 7.8 ± 3.0, 11.1 ± 1.4, and 6.8 ± 1.8, respectively.

#### 3.5.5. Effect of Eugenol on the Activity of SOD of *Sh. flexneri*

The effect of eugenol on SOD of *Sh. flexneri* was shown in Figure 9B. The activity of SOD in *Sh. flexneri* decreased when the *Sh. flexneri* was treated with different concentrations of eugenol. The activity of SOD in *Sh. flexneri* that was treated with eugenol concentrations of 1/2MIC, MIC, and 2MIC was reduced to 1.23 ± 0.09, 0.89 ± 0.09, and 0.25 ± 0.02 units, respectively (Figure 9B).

#### 3.5.6. Effect of Eugenol on the Intracellular MDA of *Sh. flexneri*

The effects of eugenol on MDA formation in *Sh. flexneri* cells were determined and shown in Figure 9C. Twenty minutes after the *Sh. flexneri* were treated by different concentrations of eugenol, the MDA content of the eugenol-treated bacteria increased and were significantly higher (*p* < 0.01) than that of the control, but there was no significant difference within the three groups.

#### 3.5.7. Effect of Eugenol on the Cell Morphology of *Sh. flexneri*

**Figure 10:**
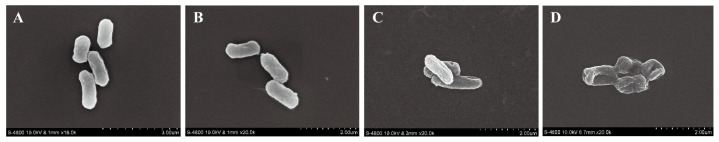
The effect of eugenol on the cell morphology of *Sh. flexneri*. (**A**) Untreated *Sh. flexneri* cells. (**B**) *Sh. flexneri* treated with eugenol at MIC. (**C**) *Sh. flexneri* cells treated with eugenol at 2MIC. (**D**) *Sh. flexneri* cells treated with eugenol at 4MIC.

FESEM was carried out to observe the changes in cell morphology. As shown in Figure 10, the untreated cells (Figure 10A) were smooth rod-shaped, while the eugenol-treated *Sh. flexneri* cells (Figure 10B–D) were wrinkled with rough surfaces. As the concentration of eugenol increased, the degree of shrinkage gradually increased from MIC to 4MIC.

## 4. Discussion

The results of the study indicated that eugenol shows an inhibitory effect against *Sh. flexneri*, and its MIC and MBC for *Sh. flexneri* are 0.5 mg/mL and 0.8 mg/mL, respectively (Figure 1). Similarly, Kang et al. studied the antibacterial activity of gallic acid against *Sh. flexneri*, and its MIC and MBC for *Sh. flexneri* were 2 mg/mL and 8 mg/mL, respectively [33]. Another study reported that the MIC and MBC of ferulic acid against *Sh. flexneri* were 2 mg/mL and 4 mg/mL [34]. Therefore, compared with the other natural products that have been reported so far, eugenol exerted stronger antibacterial activity against *Sh. flexneri*.

Outbreaks of *Sh. flexneri* have been linked to unpasteurized vegetable juices and meat in recent years [35,36]. Eugenol had a bactericidal effect on *Sh. flexneri* in LB broth and PBS (Figure 3) in this research. In order to explore its application in controlling *Sh. flexneri* in food media, minced pork and lettuce juice were selected for the experiments. In this study, regardless of whether it was the LB nutrient medium (Figure 3A), PBS non-nutrient medium (Figure 3B), lettuce juice fluid food medium (Figure 4), or pork minced solid food medium (Figure 5), it was demonstrated that eugenol greatly inactivated *Sh. flexneri*. Similarly, Ashrafudoulla et al. [37] indicated that *Vibrio parahaemolyticus* in LB broth decreased to below the detection limit after being treated with 0.4% eugenol for 10 min. The study by Cui et al. [38] showed that the number of *Listeria monocytogenes* treated by clove oil at MIC concentrations was reduced by 95.82% and 99.99% after 4 h and 8 h in the peptone yeast glucose solution. In addition, the antibacterial effects of eugenol on *Sh. flexneri* in PBS, LB, lettuce juice, and minced pork were inconsistent in this study. The strength of the inhibitory effect on *Sh. flexneri* in PBS, LB, lettuce juice, and minced pork was PBS > LB > vegetable juice > minced pork. The study by Fei et al. [39] reported that treatment with MIC olive oil polyphenol extract in normal saline non-nutrient medium and LB nutrient medium for 3 h and 10 h, respectively, reduced *Enterobacter sakazakii* concentrations below the detection limit. Differences in growth media led to different inhibitory effects. On the one hand, the difference may be related to the growth state of the bacteria, while on the other hand, this was probably due to the complex chemical composition, texture, and pH of different media affecting the antibacterial effect of eugenol against *Sh. flexneri*.

The results of our study indicated that the intracellular ROS level of *Sh. flexneri* significantly increased after eugenol treatment (Figure 9A). The study by Khan et al. [40] reported that carvacrol increased the level of ROS in *Escherichia coli* (*E. coli*) cells. Studies have shown that bacteria owned ROS removal systems, such as SOD enzyme, catalase, etc., so that ROS can maintain harmless levels in the body [41,42]. Studies have shown that the decreased liveness of SOD enzyme [30] leads to the increase in the level of intracellular ROS, which in turn leads to the inhibition of bacterial life activities. When Li et al. [30] assessed the inhibitory effect of epigallocatechin gallate on *Vibrio mimicus*, they found that epigallocatechin gallate would reduce the activity of SOD enzyme and lead to an increase in the intracellular ROS level of *Vibrio mimicus*. The intracellular SOD enzyme activity of *Sh. flexneri* treated with eugenol decreased (Figure 9B) in our study. Therefore, as an effective antibacterial method, eugenol inactivates the ROS scavenging enzymes of *Sh. flexneri*, thereby accumulating ROS in the bacteria and inducing the damage and death of *Sh. flexneri*.

The results also showed that the intracellular ROS levels of *Sh. flexneri* treated with a eugenol concentration of 2MIC were decreased compared with the intracellular ROS levels of bacteria treated with MIC eugenol (Figure 9A). This may be due to the leakage of intracellular ROS due to the damage to cell membrane integrity, resulting in the decrease of ROS at the detection level, which was similar to the findings of Liao et al. [43].

Excessive ROS in cells under oxidative stress can cause damage to important biomolecules such as lipids and proteins, thereby posing a threat to bacterial cells [44]. Polyunsaturated fatty acids in cell lipid membranes are particularly peroxidized by ROS to generate the lipid peroxidation product, MDA [45]. At 37 °C, MDA levels of *Sh. flexneri* significantly increased after eugenol treatment (*p* < 0.01) (Figure 9C). The study by Shi et al. [31] showed that the MDA content in *Escherichia coli* treated with octyl gallate was significantly increased by 1.45 times compared with the control group. Liang et al. [46] pointed out that 6% (*w*/*v*) buckwheat honey can induce *Pseudomonas aeruginosa* cells to produce malondialdehyde, indicating that membrane lipid peroxidation caused oxidative stress and led to bacterial oxidative damage. Combined with the results of the ROS experiment, as an effective antibacterial method, eugenol significantly increased the intracellular ROS level of *Sh. flexneri* (*p* < 0.01), oxidative stress occurred, and the content of malondialdehyde increased significantly (*p* < 0.01), which led to oxidative damage to the bacteria, thereby inducing the damage and death of *Sh. flexneri*.

In this study, a quantitative analysis through flow cytometry showed that eugenol treatment reduced the cell membrane integrity of *Sh. flexneri* (Figure 8). A similar study showed that 4.0 mg/mL of polysaccharides damaged the cell membrane integrity of *E. coli* [47]. It was found that the cell membranes of *Pseudomonas fluorescens* were damaged by the action of lactobionic acid at a concentration of 2MIC for 2 h [48]. Studies have shown that the excessive accumulation of ROS promotes oxidative damage to cell membranes and destroys cell membrane integrity [49]. This was the same as the results of this study (Figure 8 and Figure 9A,C), which indicated that the impaired cell membrane integrity was caused by the elevation of intracellular ROS in bacteria caused by eugenol.

The membrane potential of the bacterial cell membrane reflects the difference in potential between the inner and outer membranes of the cell and is an important indicator for measuring the permeability of the cell membrane [50]. After eugenol treatment, the membrane potential of the cell membrane was depolarized, which showed the same trend as eugenol concentration and action time within the first 15 min of processing (Figure 7). Research by Khan et al. [40] demonstrated that in carvacrol-treated *E. coli*, membrane depolarization increased, oxidation rupture increased, and bacterial cell death increased. It shows that carvacrol has a significant antibacterial effect on *E. coli*. Silverman et al. [51] reported that daptomycin caused *Staphylococcus aureus* K^+^ to be released, which led to the depolarization of the membrane potential of the cell membrane, thereby causing cell death. Therefore, it was believed that cell membrane depolarization was caused by K^+^ released to maintain membrane homeostasis, further illustrating that the cell membrane integrity of *Sh. flexneri* was damaged by eugenol.

ATP plays a vital role in life activities such as bacterial growth, replication, and survival. Intracellular ATP is necessary for functions such as the storage and supply of metabolic energy and enzyme reactions [52]. Compared with the control, the eugenol treatment significantly reduced the intracellular ATP concentration of *Sh. flexneri* (*p* < 0.01) in this study (Figure 6). Turgis et al. [53] showed that the intracellular ATP concentrations of *E. coli* O157:H7 and *Salmonella* typhi when treated with mustard essential oil were significantly decreased. The study by Guo et al. [27] showed that the intracellular ATP concentration of *Cronobacter sakazakii* treated with coenzyme Q_0_ was significantly reduced. Studies have shown that cells can change the permeability of the cell membrane through the production of plasma membrane carriers or conductance channels, thereby causing the release of ATP [52], and resulting in a decrease in the concentration of intracellular ATP. The study by Khan et al. [40] showed that carvacrol destroys the cell membrane of *E. coli*, leading to the release of ATP in the cell, and, as the concentration of carvacrol increases, the release of ATP increases. On the other hand, adverse external conditions will increase the activity of ATPase and increase the rate of ATP hydrolysis, leading to the rapid consumption of ATP in cells [52,54]. It is speculated that eugenol reduces the ATP level of *Sh. flexneri*, on the one hand, because eugenol changes the integrity of bacterial cell membranes, leading to ATP leakage, and on the other hand, because eugenol increases bacterial ATPase activity and increases ATP consumption.

In this study, the FESEM was used to observe the morphological changes of *Sh. flexneri* cells treated with eugenol (Figure 10). Based on the study by Niu et al. [55], after treatment with 0.80 mg/mL of eugenol, *E. coli* cells deformed and became irregularly shaped, with large areas of deformation and wrinkles. The study by Kang et al. [1] showed that the MIC concentration of gallic acid ruptured the cell membrane of *Sh. flexneri*, and the bacteria treated with an MBC concentration of gallic acid almost did not show normal morphology. In this study, *Sh. flexneri,* after eugenol treatment, showed atrophy in a concentration-dependent manner (Figure 10). By combining the results from the study of cell membrane integrity, severe membrane damage caused by changes in cell permeability led to the leakage of contents, thereby causing *Sh. flexneri* to shrink and become non-directional cells.

## 5. Conclusions

In this study, eugenol was confirmed to exhibit an antibacterial effect against *Sh. flexneri*. The activity of the SOD enzyme of *Sh. flexneri* was reduced by eugenol, resulting in increased intracellular ROS levels, which further led to oxidative damage to the cell membrane. The permeability and integrity of the cell membrane were disrupted by eugenol, accompanied by the leakage of intracellular ATP and the depolarization of the membrane potential. FESEM observation further confirmed the disruption of the cell membrane; all of these result in *Sh. flexneri* cell death. Eugenol also effectively inactivated *Sh. flexneri* in LB broth, PBS, minced pork, and vegetable juice. In summary, this study provided a theoretical possibility that eugenol might be used as an attractive antibacterial agent to reduce the food safety issues caused by *Sh. flexneri*.

## Data Availability

The data presented in this study are available on request from the corresponding author.
